# The Liquid-Fermentation Formulation of *Sanghuangporus sanghuang* Optimized by Response Surface Methodology and Evaluation of Biological Activity of Extracellular Polysaccharides

**DOI:** 10.3390/foods13081190

**Published:** 2024-04-13

**Authors:** Yuhan Gao, Xiaomin Li, Hui Xu, Huijuan Sun, Junli Zhang, Xiaoping Wu, Junsheng Fu

**Affiliations:** 1 College of Life Sciences, Fujian Agriculture and Forestry University, Fuzhou 350002, China; 13161198688@163.com (Y.G.); lxm2651277345@163.com (X.L.); xuhui189500@163.com (H.X.); fjwxp@126.com (X.W.); 2 Chemical and Biomolecular Engineering Department, College of Design and Engineering, National University of Singapore, Singapore 117585, Singapore; 3 Mycological Research Center, Fujian Agriculture and Forestry University, Fuzhou 350002, China; 4 Tibet Academy of Agricultural and Animal Husbandry Sciences, Lasa 850000, China; m18898002012@163.com (H.S.); xzyyxh888@163.com (J.Z.)

**Keywords:** *Sanghuangporus sanghuang*, response surface optimization, extracellular polysaccharides, biological activity

## Abstract

*Sanghuangporus sanghuang* is a rare fungus growing on mulberry trees that has immense medicinal value. This study aimed to optimize the liquid-fermentation-media formulation and culture conditions for large-scale culturing of *S. sanghuang* by performing one-way testing and response surface methodology. The antioxidant and anticancer activities of the extracellular polysaccharides from *S. sanghuang* were also analyzed. The optimal formulation and growth conditions for *S. sanghuang* were as follows: glucose, 30.2 ± 0.37 g/L; yeast extract, 14.60 ± 0.05 g/L; dandelion powder, 1.24 ± 0.01 g/L; shaker speed, 150 r/min; and temperature, 25 °C. We obtained 13.99 ± 0.42 g/L of mycelium biomass by culturing *S. sanghuang* for 15 days with the optimized formulation. This was 2-fold higher than the mycelial mass obtained with the sub-optimal formulation. The extracellular fungal polysaccharides showed significant antioxidant activity against ABTS and DPPH free radicals, and significantly reduced the in vitro growth and survival of several cancer cell lines. The anticancer activity of the extracellular fungal polysaccharides was significantly higher in the human glioma cells than in other cancer cell lines. In summary, this study optimized the liquid media formulation and conditions for the large-scale culturing of *S. sanghuang.* Furthermore, the extracellular polysaccharides from *S. sanghuang* showed significant antioxidant and anticancer activities.

## 1. Introduction

*Sanghuangporus sanghuang* is a medicinal fungus that has been used in traditional medicine for centuries in Southeast Asia [1]. *S. sanghuang* parasitizes on mulberry trees and is found in the north-eastern regions of Asia [2]. *S. sanghuang* is associated with anticancer activities [3] as well as antioxidant and anti-inflammatory properties [4]. The mycelium of *S. sanghuang* is rich in histones and improves sleep [5]. *Sanghuangporus* spp. are rich in polysaccharides, flavonoids, terpenes and other active substances. *Sanghuangporus* spp. polysaccharide is an important active substance of *Sanghuangporus* spp. Intracellular polysaccharides are mainly derived from fermentation broth [6,7,8,9]. The extracellular polysaccharides of *S. sanghuang* are widely-studied as bioactive compounds and show a broad range of pharmacological activities, including anticancer [10], hypoglycemic [11], antioxidant [12], and immunity-enhancing [13] properties. Liu et al. [14] found that *Phellinus baumii* polysaccharide can promote the proliferation of RAW264.7 cells, enhance their phagocytic ability, promote their secretion of IL-6 and TNF-α cytokines, and have immunomodulatory activity. Dong et al. [15] have shown that the crude extracellular polysaccharide (EPS) in *Phellinus igniarius* liquid fermentation broth can significantly inhibit the growth of mouse sarcoma S180 and mouse liver cancer H22.

As *S. sanghuang* has immense medicinal value, there is an increased demand for various natural products and health-improving nutritional food supplements containing *S. sanghuang*. Therefore, there is an increasing demand for culturing *S. sanghuang* on a large-scale. However, the yield of wild *S. sanghuang* is poor and insufficient to meet the medical and market demands. Although the cultivation of fungi such as *Sanghuangporus vaninii*, *Inonotus hispidus*, and *Sanghuangporus baumii* has been achieved successfully, the cultivation of *Sanghuangporus sanghuang* has remained difficult. Because the types of mulberry trees and habitats (temperature, humidity, light, oxygen concentration, etc.) that parasitize *Sanghuangporus sanghuang* are varied, their quality evaluation lacks scientificity and rigor. The current artificial cultivation technology is not yet mature, and the growth cycle is long, the yield is low, and it is susceptible to diseases and insect pests, so the cultivation of *Sanghuangporus sanghuang* is still difficult. Liquid medium is the preferred method for the large-scale cultivation of fungal mycelium because it enables full contact of the fungal clumps with the medium after inoculation and promotes the immediate absorption of nutrients. The liquid medium also enables rapid release of the fungal metabolites. Liquid culturing is simple and the formulation is flexible and amenable to adjustments. Therefore, liquid culturing is routinely used for large-scale fungal culturing and the extraction of fungal metabolites [16]. In this study, we used the one-way testing and response surface methodology to optimize the optimal carbon source, nitrogen source, and exogenous growth factors in the liquid-fermentation-media formulation for improving the yield of *S. sanghuang*. Furthermore, we performed chemical antioxidant and MTT assays to determine the antioxidant and anticancer activities of the extracellular polysaccharides of *S. sanghuang* to provide a theoretical basis for future industrial applications and drug development.

## 2. Materials and Methods

### 2.1. Materials

#### 2.1.1. Strains

Three *S. sanghuang* strains (MS-1, MS-3, and MS-7) growing on the mulberry plants were obtained from the Mycological Research Center of Fujian Agriculture and Forestry University.

#### 2.1.2. Cancer Cell Lines

HepG-2 (human liver cancer cell line), MCF-7 (human breast cancer cell line), and PC-3 (human prostate cancer cell line) cells were purchased from Saibaikang Biotechnology Co., Ltd. (Shanghai, China). HCT-116 (human colon cancer cell line) as well as T98G and U251 (human brain glioma cell lines) cells were purchased from Procell Life Technology Co., Ltd. (Wuhan, China).

#### 2.1.3. Reagents

Potato-Dextrose-Agar (PDA)-enriched medium was prepared with an extract containing 200 g potatoes (peeled), 20 g Agar, 20 g glucose, 2% agar, 5 g peptone, 1.5 g MgSO_4_, 2 g KH_2_PO_4_, 0.01 g vitamin B_1_, and up to 1 L of deionized water. The medium was sterilized at 121 °C for 30 min. The PDB-enriched medium was prepared with an extract containing 200 g potatoes (peeled), 20 g glucose, 5 g peptone, 1.5 g MgSO_4_, 2 g KH_2_PO_4_, and 0.01 g vitamin B_1_ and made up to 1 L with deionized water. The medium was sterilized at 121 °C for 30 min.

RPMI 1640 cell culture medium, DMEM cell culture medium, MEM cell culture medium, penicillin–streptomycin solution, and fetal bovine serum were used to prepare media for culturing the human cancer cell lines and stored in the refrigerator at 4 °C

PBS buffer (pH 7.4) was prepared with NaCl (8.5 g), KCl (0.2 g), Na_2_HPO_4_·12H_2_O (2.85 g), and KH_2_PO_4_ (0.27 g) in 1 L deionized water and sterilized at 121 °C for 30 min.

### 2.2. Methodology

#### 2.2.1. Strain Activation

Strain activation was performed using the method described by Ding et al. [17]. Briefly, the test tubes of seeds of MS-1, MS-3, and MS-7 strains of *Sanghuangporus sanghuang* were inoculated into the PDA enrichment medium, wrapped with a sealing film to prevent contamination, and cultured at a constant temperature of 25 °C until the mycelium completely covered the surface of the petri dish.

#### 2.2.2. Estimation of Mycelial Biomass

The mycelial biomass was estimated using the method described previously by Jia [18]. Briefly, five 7-mm-diameter fungal clusters were incubated individually in flasks containing 100 mL PDB growth medium. The cultures were grown in the dark for 15 days at 25 °C on a shaker set at 160 rpm/min. The liquid in the culture bottles was vacuum filtered to harvest the mycelia. The filtration was repeated three times. The harvested mycelia were washed with distilled water, dried in an oven at 65 °C, and weighed to record the mycelial biomass.

#### 2.2.3. The Effects of Carbon Sources on the Growth Rate of *S. sanghuang*

Glucose was replaced as the carbon source in the PDB-enrichment-medium formulation by maltose, sucrose, fructose, xylose, mannitol, mannose, lactose, or galactose (20 g/L each). The control (CK) group formulation did not have any additional carbon source. The media were inoculated with 7 mm × 7 mm × 5 mm *S. sanghuang* clumps and cultured for 15 days in the dark at 25 °C on a constant shaker set at 150 r/min. The mycelia were harvested from all of the flasks and the biomasses were estimated as described above.

#### 2.2.4. Effects of Nitrogen Sources on the Growth Rate of *S. sanghuang*

Yeast extract, peptone, ammonium tartrate, ammonium sulfate, ammonium nitrate, urea, and beef extract were used as alternative nitrogen sources in the PDB-enrichment-medium formulation. The formulation of the CK group did not have any additional nitrogen sources. The media were inoculated with 7 mm × 7 mm × 5 mm *S. sanghuang* clumps and cultivated in a shaker at 25 °C and 150 r/min for 15 days in the dark. Then, the mycelia were harvested and weighed to determine the effects of different nitrogen sources on the growth of *S. sanghuang*.

#### 2.2.5. Effects of Different Concentrations of Carbon and Nitrogen Sources on the Growth Rate of *S. sanghuang*

Based on the results of one-way carbon and nitrogen source screening, the growth rates for different strains of *Sanghuangporus sanghuang* were analyzed using different concentrations of carbon and nitrogen. Each experiment was performed with three replicates for each group. The carbon source concentrations in the media were 10, 15, 20, 25, or 30 g/L. The nitrogen source concentrations in the media were 2.5, 5, 7.5, 10, or 15 g/L. The culture media were inoculated with 7 mm × 7 mm × 5 mm *S. sanghuang* clumps and cultured at 25 °C in a shaker set at 150 rpm/min for 15 days in the dark. The mycelial biomasses were estimated as previously described.

#### 2.2.6. Effects of Exogenous Additives on the Growth of *S. sanghuang*

Previous experiments showed that dandelion powder significantly increased the growth of *S. sanghuang* mycelium on the plate. This suggested that dandelion powder acted as a growth factor for *S. sanghuang*. Therefore, we added 0, 0.5, 0.75, 1, 1.25, or 1.5 g/L of dandelion powder to the PDB-enriched medium, inoculated the media with 7 mm × 7 mm × 5 mm *S. sanghuang* clumps, and cultured them on a shaker at 25 °C and 150 r/min in the dark for 15 days. The mycelium biomass was estimated as previously described.

#### 2.2.7. Response Surface Methodology for Optimizing the *S. sanghuang* Growth Medium Formulation

The response surface methodology (RSM) was used to optimize the concentrations of different carbon and nitrogen sources and other additional factors for growing *S. sanghuang*. The Box–Behnken design principle and Design Expert 11 software (ver. 11) were used to perform three levels of response surface experiments to determine the optimal concentrations of the carbon source, nitrogen source, and dandelion powder for obtaining the maximum mycelial biomass. Table 1 shows the concentrations of different factors used in the media formulations and levels of the response surface experiments.

Accuracy of the RSM prediction results was verified by liquid-fermentation-media culturing of the fungus with the best-predicted formulation. The mycelia were collected, dried and weighed. The weight of the mycelia was compared with that obtained under sub-optimal conditions.

#### 2.2.8. Extraction of Extracellular Polysaccharides

The fermentation liquid was collected after growing the fungus, vacuum filtered thrice, and centrifuged at 10,000 rpm for 3 min to remove the mycelial remnants. Then, the fermentation liquid was concentrated by spin distillation to 1/5th of the original volume. Subsequently, ethanol and concentrated fermentation liquid were mixed in a 1:4 ratio by volume. Alcohol precipitation was performed overnight at 4 °C. The precipitate was harvested after centrifugation and re-solubilized with water. We added Sevag reagent (chloroform:n-butanol volume ratio is 4:1) into the crude polysaccharide solution, shook it thoroughly and centrifuged at 5000 rpm/min for 5 min, and then mixed the water phase with chloroform for separation. We added an equal volume of Sevag reagent to the water phase and repeated several times until there was no obvious protein layer. Then, the protein-free solution was incubated with ether and methanol to remove the impurities. Finally, the crude polysaccharide solution was dialyzed in running water for 48 h, freeze-dried, and stored at −20 °C.

#### 2.2.9. Chemical Antioxidant Activity of the Extracellular Polysaccharides

An ABTS free radical scavenging assay was performed using the protocol described by Li et al. [19] with slight modifications. Different concentrations of the extracellular polysaccharide and vitamin C(VC) solutions (0.025, 0.05, 0.25, 0.5, 1, 2, and 5 mg/mL) were prepared and added into wells of a 96-well plate. Then, an equal volume of the ABTS solution (7 mM) was added into each well. In the blank group, an equal volume of distilled water was added instead of the polysaccharide solution. The reaction was performed at 25 °C. Absorbance was measured at 734 nm in an enzyme-labeling instrument. The antioxidant activity was calculated using the average value from five replicates for each concentration of the extracellular polysaccharide solution and a calibration curve of VC with the following formula:

ABTS free radical scavenging rate (%) = [1 − (A_x_ − A_x0_)/A_0_] × 100,

where A_x_ is the absorbance value for the experimental group, A_0_ is the absorbance value for the blank group, and A_x0_ is the absorbance value for the control group.

Assessment of DPPH radical scavenging activity was performed according to the method described by Luo et al. [20] with slight modifications. Different concentrations (0.025, 0.05, 0.25, 0.5, 1, 2, and 5 mg/mL) of the extracellular polysaccharide and VC solutions were prepared and aliquoted into 96-well plates. Then, an equal volume of the DPPH solution (0.2 mM of 80% ethanol solution) was added into each well. In the blank group, an equal volume of anhydrous ethanol was used instead of the polysaccharide solution. The plate was incubated for 20 min at 25 °C. Then, absorbance was measured at 517 nm using an enzyme-labeling instrument. DPPH radical scavenging activity was calculated using VC as the positive control group according to the following formula:

DPPH radical scavenging rate (%) = [1 − (A_x_ − A_x0_)/A_0_] × 100,

where A_x_ is the absorbance value of the experimental group, A_0_ is the absorbance value of the blank group, and A_x0_ is the absorbance value of the control group.

#### 2.2.10. Ferric Reducing Antioxidant Power (FRAP) Assay

The FRAP assay was performed according to the method described by Benzie et al. [21] with slight modifications. Different concentrations (0.025, 0.05, 0.25, 0.5, 1, 2, and 5 mg/mL) of the extracellular polysaccharides were prepared. Then, 30 μL aliquots of the polysaccharide solutions were added to each well of a 96-well plate and incubated with 180 μL of FRAP solution for 20 min at 25 °C. The blank group contained 30 μL of deionized water and 180 μL of FRAP solution. A standard curve was prepared by incubating 30 μL aliquots of 0.025, 0.1, 0.2, 0.4, 0.8, and 1 mmol/L FeSO_4_ solutions with 180 μL of FRAP solution at 25 °C for 20 min. Absorbance was measured at 593 nm in enzyme-labeling instrument. Five replicates were used for each group. The average value was calculated using the following formula:

FRAP value = A_x_ − A_0_ − A_x0_,

where A_x_ is the absorbance value of the experimental group, A_0_ is the absorbance value of the blank group, and A_x0_ is the absorbance value of the control group.

#### 2.2.11. Effects of Extracellular Polysaccharides on Cancer Cell Proliferation

Based on the protocol described by Zhang et al. [22] with slight modifications, cancer cells were cultured in cell culture medium containing 10% fetal bovine serum and 1% penicillin/streptomycin mixture in a humidified incubator at 37 °C and 5% CO_2_. Then, 200 μL of cancer cells (HepG-2, MCF-7, PC-3, HCT-116, U251, and T98G) in the logarithmic phase of growth were seeded in the 96-well plates and cultured for 24 h. The culture medium was removed after observing adherence of cells. In the experimental group, the cancer cells were incubated with 200 μL of cell culture medium containing different concentrations of three extracellular polysaccharides (0.025, 0.05, 0.25, 0.5, 1, 2 and 5 mg/mL). We used five replicates for each group. The blank group wells contained 200 μL of cell culture medium without any cells. In the control (CK) group, the cells were cultured with the medium for 24 h. Subsequently, after removing the medium, we added 10 μL of 5 mg/mL MTT solution and 90 μL of cell culture solution to each well and incubated for 4 h. Then, the culture solution was removed from all the wells carefully. We then added 150 μL of DMSO to each well. After mixing on a shaker for 15 min, the absorbance value (OD value) of the solution was determined at a wavelength of 490 nm in an enzyme-labeling instrument.

### 2.3. Statistical Analysis

SPSS 25.0 statistical software was used for statistical analysis. The data were expressed as mean ± standard deviation (x ± s). The t-test was used to determine statistically significant differences between two groups of data, whereas ANOVA was used to compare the data between multiple groups. *p* < 0.05 was considered statistically significant. *p* < 0.01 was considered highly statistically significant. 

## 3. Results

### 3.1. Identification of an Optimal Carbon Source for Culturing S. sanghuang

**Figure 1:**
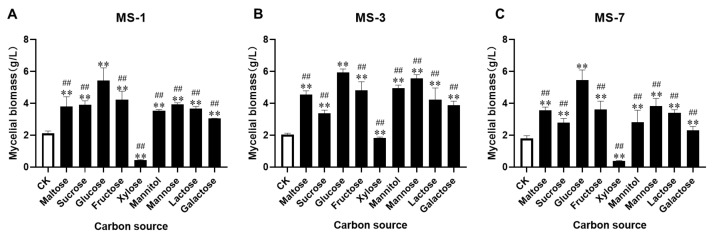
The effects of different carbon sources on the growth of strains of *S. sanghuang*. (**A**) MS-1, (**B**) MS-3, and (**C**) MS-7. Note: ** represents *p* < 0.01 for comparisons with the CK group; ^##^ represents *p* < 0.01 for comparisons with the optimal carbon source.

Firstly, we investigated the effects of different carbon sources in the PDB-enrichment medium, including maltose, sucrose, glucose, fructose, xylose, mannitol, mannose, lactose, and galactose, on the growth of *S. sanghuang*. As shown in Figure 1, the effects of different carbon sources on the mycelium biomass (high biomass to low) of *S. sanghuang* were as follows: glucose > mannose > fructose > maltose > mannitol > lactose > sucrose > galactose > CK > xylose. The three strains of *S. sanghuang* showed similar carbon source requirements. Glucose was the most effective and xylose was the least effective carbon source. Therefore, glucose was selected as the carbon source for culturing *S. sanghuang* using the liquid fermentation medium.

### 3.2. Identification of an Optimal Nitrogen Source for Culturing S. sanghuang

**Figure 2:**
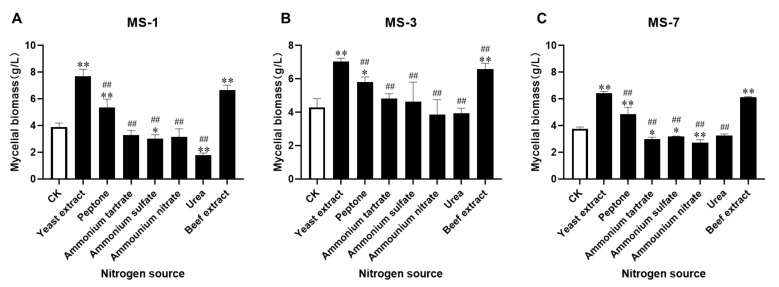
The effects of different nitrogen sources on the growth of *S. sanghuang*. (**A**) MS-1; (**B**) MS-3; (**C**) MS-7. Note: * represents *p* < 0.05 and ** represents *p* < 0.01 for comparisons with the CK group; ^##^ represents *p* < 0.01 for comparisons with the optimal nitrogen source.

Next, we investigated the effects of different nitrogen sources in PDB-enrichment medium on the growth of *S. sanghuang*. As shown in Figure 2, the effects of different nitrogen sources (high biomass to low) on the mycelium biomass of *S. sanghuang* were as follows: yeast extract > beef extract > peptone > CK > ammonium tartrate > ammonium sulfate > ammonium nitrate > urea. The requirement for a nitrogen source was high for all three strains of *S. sanghuang*. Yeast extract was the most effective nitrogen source, whereas ammonium tartrate, ammonium sulfate, ammonium nitrate, and urea were the least effective nitrogen sources. Therefore, yeast extract was selected as the nitrogen source for the liquid fermentation medium used to grow *S. sanghuang*.

### 3.3. Optimizing the Concentrations of the Carbon and Nitrogen Sources and the Growth Factors for Culturing S. sanghuang

#### 3.3.1. Optimizing the Glucose Concentration for Culturing *S. sanghuang*

**Figure 3:**
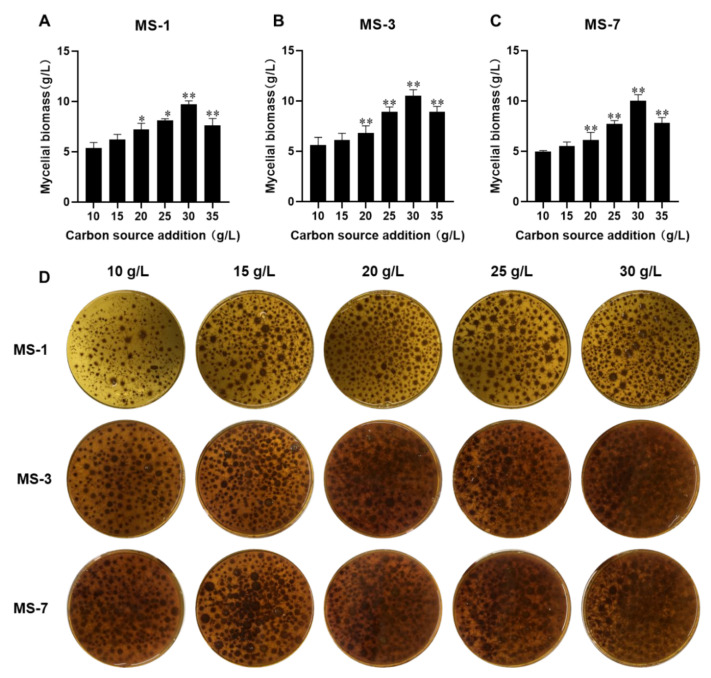
The effects of different concentrations of glucose on the growth of *S. sanghuang*. The mycelial biomasses of (**A**) MS-1; (**B**) MS-3; and (**C**) MS-7. (**D**) Fermentation liquid media with different glucose concentrations for growing MS-1, MS-3, and MS-7 strains of *S. sanghuang*. Note: * represents *p* < 0.05 and ** represents *p* < 0.01 when compared with the previous group.

As shown in Figure 3, PDB-enrichment medium with 30 g/L glucose showed the maximal and fastest growth of *S. sanghuang*. The liquid culture medium with 30 g/L glucose showed the optimal growth of *S. sanghuang*. The mycelial growth of *S. sanghuang* was sub-optimal when the glucose concentration was <30 g/L or >30 g/L.

#### 3.3.2. Optimizing the Yeast Extract Concentration for Culturing *S. sanghuang*

**Figure 4:**
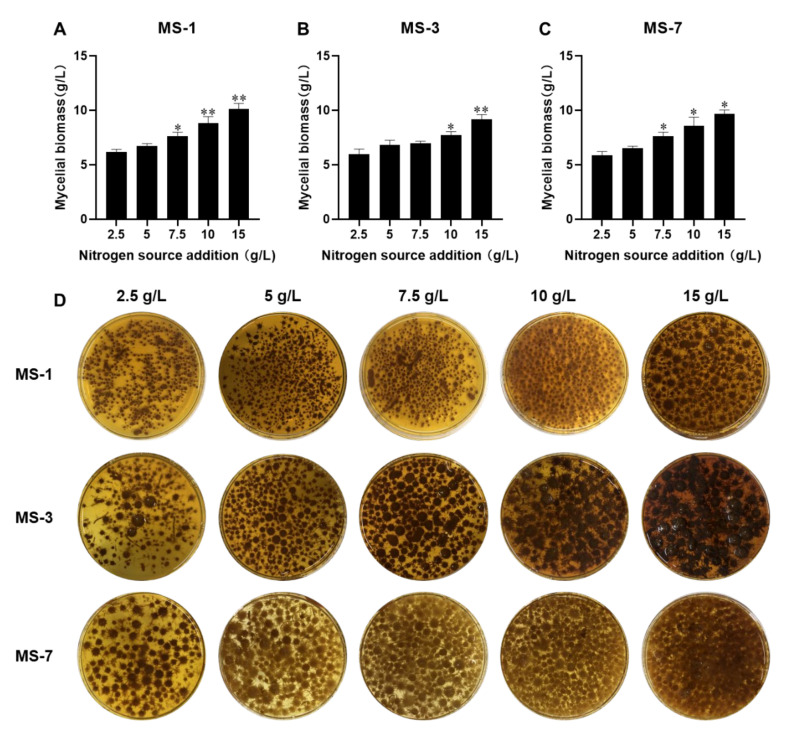
The effects of different yeast extract concentrations on the growth of *S. sanghuang*. The mycelial biomasses of (**A**) MS-1; (**B**) MS-3; and (**C**) MS-7 strains of *S. sanghuang*. (**D**) Fermentation liquid media with different yeast extract concentrations for growing MS-1, MS-3, and MS-7 strains of *S. sanghuang*. Note: * represents *p* < 0.05 and ** represents *p* < 0.01 compared with the previous group.

As shown in Figure 4, the mycelial growth rate and biomass of *S. sanghuang* increased proportionately with increasing concentrations of yeast extract in the growth medium and they reached maximums at a concentration of 15 g/L yeast extract. The solubility of the yeast extract was also maximal at 15 g/L. Therefore, 15 g/L yeast extract was selected for the growth medium of *S. sanghuang*.

#### 3.3.3. Optimizing the Growth Factor Concentration for Culturing *S. sanghuang*

**Figure 5:**
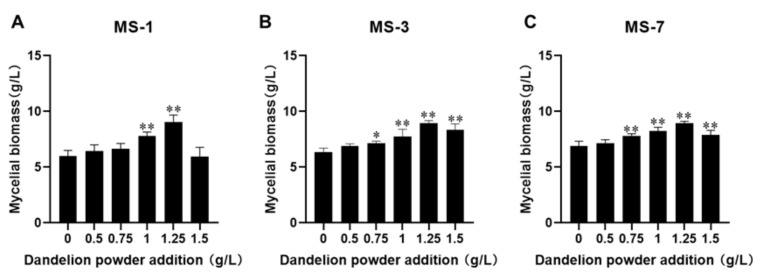
The effects of dandelion powder on the growth of strains of *S. sanghuang*. (**A**) MS-1; (**B**) MS-3; (**C**) MS-7. Note: * represents *p* < 0.05 and ** represents *p* < 0.01 compared with the CK group.

After optimizing the concentrations of the carbon and nitrogen sources, we investigated the effects of dandelion powder as an exogenous additive to improve the growth of *S. sanghuang*. As shown in Figure 5, the optimal concentration of dandelion powder for the maximal growth of *S. sanghuang* was 1.25 g/L. The mycelial biomass for the three strains of *S. sanghuang* grown with media containing 1.25 g/L dandelion powder was 1.29~1.5 fold higher than the blank group. The mycelial growth increased gradually, peaked in media containing 1.25 g/L dandelion powder, and subsequently declined in media with higher concentrations of dandelion powder (>1.25 g/L). These results showed that higher concentrations of dandelion powder suppressed the growth of *S. sanghuang*. Therefore, the optimal concentration of dandelion powder in the growth medium for *S. sanghuang* was 1.25 g/L.

### 3.4. Response Surface Methodology Results

#### 3.4.1. Box–Behnken’s Central Combination Design Results

Response surface analysis tests were performed with the concentrations of glucose, yeast extract, and dandelion powder as independent variables and the mycelial biomass of *S. sanghuang* as the response value. The experimental scheme and the results are shown in Table 2, Table 3 and Table 4.

#### 3.4.2. Multiple Quadratic Regression Fitting and ANOVA

Next, we performed experiments using the Box–Behnken’s central combination design. Then, using Design Expert software (ver. 11), we performed regression analysis of the mycelial biomass for different growth conditions and compared the data by analysis of variance (ANOVA) (Table 5, Table 6 and Table 7).

As shown in Table 4, Table 5 and Table 6 models with *p* < 0.05 and R^2^ > 0.98 as a coefficient of determination were considered to be well fitted for optimal mycelial growth. If the *p*-value was >0.05, then the model was not well fitted, and this suggested that the original model was more accurate. Therefore, the model was used to analyze the experimental results and predict the response values. The following multiple regression equations were obtained for the mycelial biomass of *Sanghuangporus sanghuang* (Y) based on independent variables such as glucose (A), yeast extract (B), and dandelion powder (C):

Y_MS-1_ =13.828 + 0.395A − 0.8675B − 0.2625C + 0.15AB + 0.31AC + 0.615BC − 1.9015A^2^ − 2.4765B^2^ − 3.0365C^2^

Y_MS-3_ = 13.6 + 0.31375A − 0.6075B − 0.32125C + 0.2925AB + 0.15AC + 0.4925BC − 2.045A^2^ − 2.2025B^2^ − 2.81C^2^

Y_MS-7_ = 13.94 − 0.08375A − 0.98B − 0.19625C + 0.075AB − 0.3575AC + 0.715BC − 1.50375A^2^ − 2.81125B^2^ − 2.91375C^2^

#### 3.4.3. Significance Test Results

To test the validity of the multiple regression equations, the mathematical model for the estimated mycelium biomass of *S. sanghuang* was evaluated by ANOVA and the partial regression coefficients of the factors were analyzed (Table 5, Table 6 and Table 7). In the primary term, the regression coefficient B was highly significant. This suggested that the concentration of the yeast extract significantly influenced the growth of *S. sanghuang*. In the secondary term, the partial regression coefficients A, B, and C reached a level of high significance. The regression coefficient of the interaction term BC was more significant than those of the other factors. This suggested that the interaction between yeast extract and dandelion powder significantly influenced the growth of *S. sanghuang*. The out-of-fit term was not significant, and the model was appropriate. The goodness-of-fit R value was > 0.98, which was close to the real data point and suggested good fitting of the equation to the experimental set-up.

#### 3.4.4. Response Surface Analysis of Two-by-Two Interactions of Factors Affecting the Culturing of *S. sanghuang*

Based on the regression equation, we performed response surface analysis to determine the effects of media containing different amounts of glucose, yeast extract, and dandelion powder on the growth of *S. sanghuang*.

**Figure 6:**
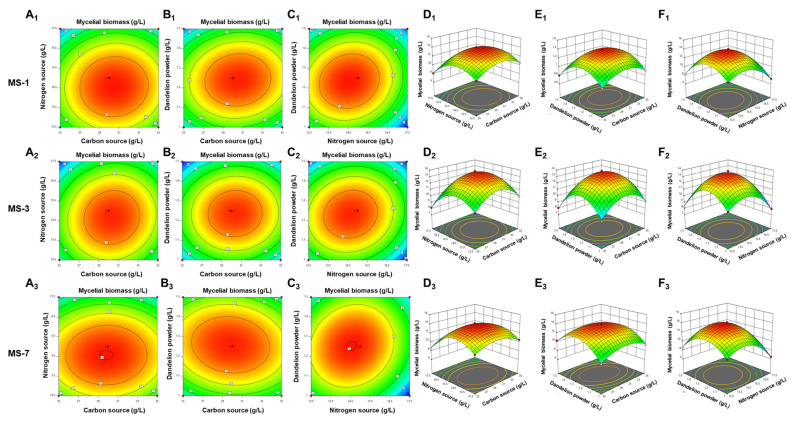
Response surface stereograms based on the Box–Behnken design for the optimization of liquid-fermentation-media components for growing *S. sanghuang*. ((**A1**–**A3**)–(**C1**–**C3**)) Corresponding contour values. (**D1**–**D3**) Interactions between different concentrations of carbon and nitrogen sources. (**E1**–**E3**) Interactions between different concentrations of the carbon source and the dandelion powder. (**F1**–**F3**) Interactions between different concentrations of the nitrogen source and the dandelion powder.

As shown in Figure 6, the optimal concentrations of yeast extract and dandelion powder in the enrichment medium were necessary for the maximal growth of *S. sanghuang*. Therefore, according to results shown in Figure 6 and the ANOVA data, the order of effects for the growth of *S. sanghuang* were as follows: BC > AC > AB. The first-order partial derivatives of the quadratic regression equation were used for the analysis. The concentrations of different factors for obtaining the maximum response value (Y) (*S. sanghuang* biomass) were as follows: (1) the theoretical mycelial biomass for the MS-1 strain was 13.93 g/L when the medium contained 30.46 g/L of the carbon source, 14.55 g/L of the nitrogen source, and 1.24 g/L of dandelion powder; (2) the theoretical mycelial biomass for the MS-3 strain was 13.66 g/L when the medium contained 30.32 g/L of the carbon source, 14.64 g/L of the nitrogen source, and 1.23 g/L of the dandelion powder; and (3) the theoretical mycelial biomass for the MS-3 strain was 14.03 g/L when the medium contained 29.83 g/L of the carbon source, 14.57 g/L of the nitrogen source, and 1.25 g/L of the dandelion powder.

#### 3.4.5. Validation of Fit Optimization

**Figure 7:**
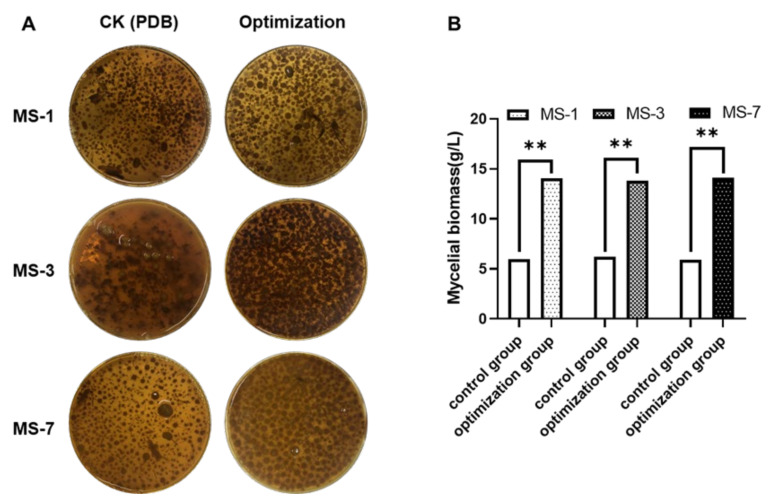
Validation of the optimized liquid fermentation formulation for growing *S. sanghuang*. (**A**) The CK group (PDB) and optimized-fermentation-liquid-growth formulations for *S. sanghuang*. (**B**) The mycelial biomass for the MS-1, MS-3, and MS-7 strains of *S. sanghuang* with the control and optimized formulations. Note: ** represents *p* < 0.01 compared with the CK (PDB) group.

To verify the accuracy of the model prediction, we performed a validation test with three sets of replicates using optimized growth medium components based on the response surface test. The results of the validation test are shown in Figure 7. The mycelium yield for the MS-1 strain with the optimized formulation was 14.0 4 ± 0.61 g/L. This was 2.36 times higher than the sub-optimal formulation (5.94 ± 0.305 g/L) and slightly higher than the predicted value (13.93 g/L). The mycelial yield for the MS-3 strain with the optimized formulation was 13.82 ± 0.3 g/L. This was 2.21 fold higher than the sub-optimal formulation (6.23 ± 0.305 g/L) and slightly higher than the predicted value (13.66 g/L). The mycelial yield for the MS-7 strain with the optimized formulation was 14.12 ± 0.31 g/L. This was 2.39 times higher than the sub-optimal formulation (5.9 ± 0.3 g/L) and slightly greater than the predicted value (14.03 g/L). These results confirmed the goodness-of-fit between the model and the actual values. Therefore, the fermentation medium formulation that had been optimized according to the response surface test method provided the necessary theoretical and scientific basis for the commercial cultivation of *S. sanghuang* in the future.

### 3.5. Antioxidant Activity of Extracellular Fungal Polysaccharides

**Figure 8:**
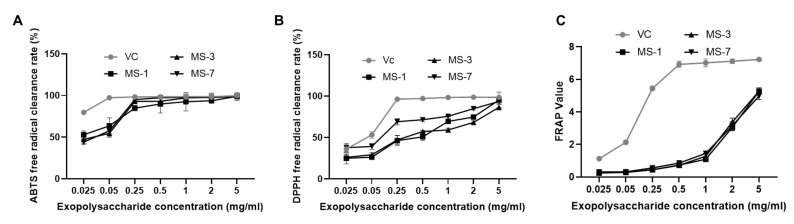
Antioxidant activities of the polysaccharides extracted from *S. sanghuang*. (**A**) ABTS-radical scavenging capacity of *S. sanghuang*. (**B**) DPPH radical scavenging capacity of the polysaccharides extracted from *S. sanghuang*. (**C**) FRAP assay results show the total antioxidant capacity of the polysaccharides extracted from *S. sanghuang*.

As shown in Figure 8, all three strains of *S. sanghuang* showed strong antioxidant activity. The *S. sanghuang* extracellular polysaccharides showed significantly high ABTS free radical scavenging activity that was comparable with the Vc control group, but the DPPH free radical scavenging activity of the *S. sanghuang* extracellular polysaccharides was about 50% lower than that of the Vc control group. The total antioxidant capacity of the *S. sanghuang* extracellular polysaccharides was weak at lower concentrations but increased rapidly at higher concentrations and was comparable with the Vc control group.

The three strains of *S. sanghuang* showed differences in their scavenging ability for the two free radicals. In the ABTS free radical scavenging experiments, the MS-1 strain showed the strongest scavenging ability (EC_50_ = 0.02 mg/mL) followed by the MS-3 strain (EC_50_ = 0.03 mg/mL) and the MS-7 strain (EC_50_ = 0.032 mg/mL). In the DPPH free radical scavenging experiment, the MS-7 strain showed the strongest scavenging ability (EC_50_ = 0.08 ± 0.00229 mg/mL) followed by the MS-1 strain (EC_50_ = 0.273 ± 0.0071 mg/mL) and the MS-3 strain (EC_50_ = 0.291 ± 0.0025 mg/mL). The results of the FRAP assay demonstrated that the total antioxidant capacity of the *S. sanghuang* extracellular polysaccharide was significantly high. The maximum FRAP values were 5.26 ± 0.11, 5.27 ± 0.19, and 4.97 ± 0.22 for the MS-1, MS-3, and MS-7 strains, respectively, when the polysaccharide concentration was 5 mg/mL.

### 3.6. Evaluation of the Anticancer Activity of the Extracellular Fungal Polysaccharides from S. sanghuang

**Figure 9:**
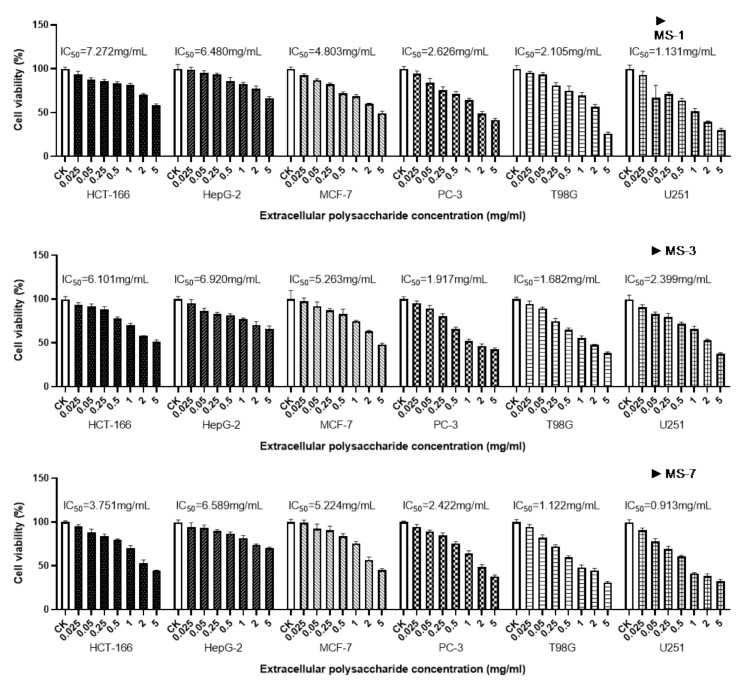
MTT assay results show the effects of the polysaccharides extracted from *S. sanghuang* on the growth of various cancer cells.

Next, we analyzed the in vitro anticancer activities of the *S. sanghuang* extracellular polysaccharides on six types of cancer cells. The extracellular polysaccharides of *S. sanghuang* inhibited the growth of all six types of cancer cell lines in the following order (high to low inhibitory effect): U251 > T98G > PC-3 > HCT-116 > MCF-7 > HepG-2 (Figure 9).

The extracellular polysaccharides of *S. sanghuang* showed the strongest inhibitory effects in the U251 human glioma cells. Furthermore, extracellular polysaccharides of the MS-7 strain (half inhibitory concentration of IC_50_ = 1.56 ± 0.96 mg/mL) showed higher anticancer efficacy than those from the MS-1 and MS-3 strains. The extracellular polysaccharides of *S. sanghuang* also significantly inhibited growth of human glioblastoma T98G cells. The extracellular polysaccharides of the MS-1 strain significantly inhibited the growth of T98G cells (*p* < 0.01) with an IC_50_ of 1.66 ± 0.44 mg/mL. The extracellular polysaccharides of *S. sanghuang* showed higher pro-apoptotic effects on the PC-3 prostate cancer cells. The MS-3 strain showed stronger pro-apoptotic effects than the other two strains (IC_50_ = 1.917 mg/mL). *S. sanghuang* showed weaker anticancer efficacy on the colon cancer cell line, HCT-116. Furthermore, the MS-7 strain showed higher anticancer activity on the HCT-116 cells than did the MS-1 and MS-3 strains, with an IC_50_ value of 3.751 mg/mL. The apoptosis rate was 44.33% in the HCT-116 cells after 24 h of treatment with the highest concentration of the MS-7 polysaccharides. At the highest concentrations, the anticancer activities of the MS-1 and MS-3 polysaccharides were weaker in the HCT-116 cells, with survival rates of 58.35% and 51.52%, respectively. The pro-apoptotic effects of the *S. sanghuang* polysaccharides were stronger on the breast cancer MCF-7 cells, with a survival rate ranging from 45.38% to 49.20% at polysaccharide concentrations of 5 mg/mL. The pro-apoptotic effects of *S. sanghuang* extracellular polysaccharides in the HepG-2 (hepatocellular carcinoma cells) cells were poor. At the 5 mg/mL polysaccharide concentration, the survival rate of the HepG2 cells ranged between 66.04% and 69.89%. Moreover, the IC_50_ values were comparable in the HepG-2 cells for the polysaccharides from the MS-1 (6.48 mg/mL), MS-3 (6.92 mg/mL), and MS-7 (6.589 mg/mL) strains.

## 4. Discussion

*Sanghuangporus* spp. is a rare medicinal fungus. Polysaccharide is an important active substance of its fruiting body and mycelium; it is a type of compound with hypoglycemic, hypolipidemic, antioxidant, anti-tumor, and immunomodulatory activities [22]. *Sanghuangporus* spp. is a perennial fungus. Since the growth of wild *S. sanghuang* fruiting bodies is limited by natural conditions and has a long growth cycle, its output cannot meet market demand. At present, the cultivation scale of *S. sanghuang* is small and unevenly distributed, and the planting technology is immature, making it difficult to guarantee the yield and quality of *S. sanghuang*, and it is difficult to increase the added value of *S. sanghuang* products [23]. There is a large talent gap in the resource evaluation, standard formulation, improved variety selection, and technology promotion of *S. sanghuang*, which restricts the high-quality development of a *S. sanghuang* industry [23]. Therefore, the artificial cultivation technology of *S. sanghuang* is still in the bottleneck period, and liquid fermentation culture of *S. sanghuang* has become another research hotspot. Liquid fermentation culture can quickly obtain a large amount of metabolites and can directly extract physiologically active substances for medicinal research. Compared with solid fermentation, liquid fermentation has the advantages of continuous production, low cost, simple operation, short fermentation time, high yield, and high efficiency.

Therefore, development of the optimal liquid-fermentation-media formulation and conditions were necessary for the large-scale production of *S. sanghuang*. In this study, we comprehensively analyzed the effects of different carbon and nitrogen sources, as well as exogenous growth factors, to develop the optimal liquid-fermentation-media formulation for maximal mycelial yield. We also performed the response surface method optimization test using a Box–Behnken design to analyze interactions between various factors. Furthermore, we analyzed the antioxidant and anticancer activities of the extracellular polysaccharides from three strains of *S. sanghuang*. Previous studies have shown that, although different strains of *S. sanghuang* show similar morphology, their requirements for carbon, nitrogen, and growth factors vary considerably. Our data showed that glucose was the optimal carbon source for the liquid fermentation medium used to grow *S. sanghuang.* This was comparable with the composition of the PDA medium that was used to grow *S. Sanghuang* in a previous study [24]. Jiang et al. also reported that glucose was the most significant requirement for the growth of *S. sanghuang* [25]. Furthermore, our data showed that yeast extract was the most suitable nitrogen source for growing *S. Sanghuang.* Organic nitrogen sources such as yeast extract, beef extract, and peptone were favorable for the optimal growth of *S. sanghuang*, but inorganic nitrogen sources such as ammonium tartrate, ammonium sulfate, ammonium nitrate, and urea were unfavorable for the growth of *S. sanghuang*. These results were comparable with the findings reported by Cheng et al. [11]. Since the nutritional conditions significantly alter the growth of *S. sanghuang*, a comprehensive study of nutrients and growth factors was necessary to develop an efficient, affordable, and optimal culture medium for obtaining maximal mycelial yield. Many researchers have chosen to add growth factors of natural components to promote the growth or the production of bioactive compounds of *S. sanghuang*. For example, based on the study of *Sanghuangporus baumii* medium of *Morus alba* L., Wang et al. reported that a mulberry bark extract promoted the growth of *S. sanghuang* [26]. In this study, 1.25 g/L dandelion powder significantly promoted the growth of *S. sanghuang*.

Furthermore, we analyzed the antioxidant and anticancer activities of the extracellular polysaccharides from *S. sanghuang* by in vitro experiments. The polysaccharides from *S. sanghuang* showed stronger ABTS-radical scavenging ability than DPPH radical scavenging ability. The total antioxidant capacity was also stronger at higher concentrations of the polysaccharides. When the concentration of extracellular polysaccharide is 0.5 mg/mL, the scavenging rate of ABTS free radicals by extracellular polysaccharide can reach 90% and above, which shows that the antioxidant capacity of *S. sanghuang* extracellular polysaccharides is substantial. Within a certain concentration range, increasing the polysaccharide concentration can improve the scavenging ability of ABTS and DPPH free radicals. The antioxidant effects of the exopolysaccharide of the *S. sanghuang* can provide a theoretical basis for its application in medical dressings, facial cleansers, facial masks, antioxidants, etc. *S. sanghuang* also has triterpenoids, flavonoids, and other compounds, but its biological activity needs to be further explored. Lang et al. [27] found that *Phellinus baumii* flavonoids have strong reducing power and strong scavenging ability for DPPH free radicals and hydroxyl free radicals, and the antioxidant capacity is dose-dependent on the flavonoid concentration. Li [28] reported that the flavonoid compounds of sanghuang had strong DPPH free radical scavenging ability and iron reduction ability.

MTT assay results showed that the polysaccharides from *S. sanghuang* significantly decreased the survival rate of U251 and T98G cells (human glioma cells), and PC-3 cells (human prostate cancer cells). The anticancer effects of the polysaccharides from *S. sanghuang* were poorer on the remaining three types of cancer cells. Therefore, in future studies, further investigations are necessary to determine the mechanisms by which the extracellular polysaccharides of *S. sanghuang* inhibit the growth and survival of cancer cells. The unique anticancer effects of sanghuang polysaccharides has made this a hot topic in functional food research at home and abroad. Liu et al. [14] have shown that *Phellinus baumii* polysaccharides can inhibit the proliferation of Hela and SGC-7901 cells by hindering cell division. How *S. sanghuang* extracellular polysaccharides exert anticancer effects remains to be further studied in later trials. Guo’s [29] research found that *S. sanghuang* triterpenoids have inhibitory effects on PC-3, HepG-2, MDA-MB-231, HCT-116, and other cancer cells, and that they have a certain anticancer potential. In the future, we may measure the metabolic profile of *S. sanghuang* and analysis its correlation with biological activity.

## 5. Conclusions

The optimal formula for liquid fermentation of *S. sanghuang* obtained after optimization in this study is as follows: glucose 30.2 ± 0.33 g/L, yeast extract 14.58 ± 0.05 g/L, and dandelion powder 1.24 ± 0.01 g/L; under these conditions, the mycelium biomass can reach 13.99 ± 0.42 g/L, which is 2-fold higher than the formula used before optimization. Further research found that the extracellular polysaccharides from *S. sanghuang* showed high in vitro antioxidant activity against ABTS and DPPH radicals as well as strong total antioxidant capacity; at the same time, the extracellular polysaccharide of *S. sanghuang* has a good inhibitory effect on the tested cancer cells. Among them, U251 and T98G cells are more sensitive. This study shows that the exopolysaccharides of *S. sanghuang* have good antioxidant activity and anticancer activity.

## Figures and Tables

**Table 1:** Factors and levels of Box-Behnken test of *Sanghuangporus sanghuang*.

Level		Factor	
	A: Carbon Source (g/L)	B: Nitrogen Source (g/L)	C: Dandelion Powder (g/L)
−1	25	12.5	1
0	30	15	1.25
1	35	17.5	1.5

**Table 2:** Response surface design and test results of MS-1.

No.	A: Carbon Source (g/L)	B: Nitrogen Source (g/L)	C: Dandelion Powder (g/L)	Mycelial Biomass (g/L)
1	25	17.5	1.25	8.1
2	25	15	1	8.9
3	35	15	1.5	9.5
4	30	15	1.25	14.06
5	30	17.5	1	7.2
6	30	15	1.25	13.83
7	35	17.5	1.25	9.1
8	25	15	1.5	8
9	30	12.5	1.5	8.2
10	30	12.5	1	10.2
11	35	12.5	1.25	10.5
12	30	15	1.25	13.76
13	30	15	1.25	13.46
14	30	17.5	1.5	7.66
15	30	15	1.25	14.03
16	35	15	1	9.16
17	25	12.5	1.25	10.1

**Table 3:** Response surface design and test results of MS-3.

No.	A: Carbon Source (g/L)	B: Nitrogen Source (g/L)	C: Dandelion Powder (g/L)	Mycelial Biomass (g/L)
1	30	12.5	1	10.13
2	25	12.5	1.25	9.96
3	25	15	1	8.76
4	30	15	1.25	13.86
5	30	12.5	1.5	8.26
6	30	15	1.25	13.56
7	35	17.5	1.25	9.33
8	25	17.5	1.25	8.16
9	30	15	1.25	13.86
10	30	17.5	1.5	8.03
11	25	15	1.5	8.06
12	35	12.5	1.25	9.96
13	30	15	1.25	13.26
14	30	17.5	1	7.93
15	35	15	1.5	9.03
16	30	15	1.25	13.46
17	35	15	1	9.13

**Table 4:** Response surface design and test results of MS-7.

No.	A: Carbon Source (g/L)	B: Nitrogen Source (g/L)	C: Dandelion Powder (g/L)	Mycelial Biomass (g/L)
1	25	15	1	8.93
2	30	12.5	1.5	8.5
3	35	17.5	1.25	8.6
4	30	15	1.25	13.83
5	35	15	1.5	9.4
6	30	15	1.25	14.17
7	25	12.5	1.25	10.8
8	30	17.5	1.5	8.06
9	30	17.5	1	6.5
10	30	15	1.25	14
11	30	12.5	1	9.8
12	30	15	1.25	13.8
13	25	17.5	1.25	8.6
14	35	15	1	9.46
15	35	12.5	1.25	10.5
16	25	15	1.5	10.3
17	30	15	1.25	13.9

**Table 5:** Variance analysis and significance tests of MS-1.

Source	Sum of Squares	Df	Mean Squares	F-Value	*p*-Value
Model	98.62	9	10.96	205.43	<0.0001
A	1.25	1	1.25	23.4	0.0019
B	6.02	1	6.02	112.87	<0.0001
C	0.5512	1	0.5512	10.33	0.0148
AB	0.09	1	0.09	1.69	0.2351
AC	0.3844	1	0.3844	7.21	0.0313
BC	1.51	1	1.51	28.36	0.0011
A2	15.22	1	15.22	285.41	<0.0001
B2	25.82	1	25.82	484.13	<0.0001
C2	38.82	1	38.82	727.83	<0.0001
Residual	0.3734	7	0.0533	0.788	0.5601
Lack of fit	0.1387	3	0.0462		
Pure error	0.2347	4	0.0587		
Cor Total	98.99	16			
R^2^ = 0.9962; Adjusted R^2^ = 0.9914; Predicted R^2^ = 0.9739					

**Table 6:** Variance analysis and significance tests of MS-3.

Source	Sum of Squares	Df	Mean Squares	F-Value	*p*-Value
Model	85.4	9	9.49	168.92	<0.0001
A	0.7875	1	0.7875	14.02	0.0072
B	2.95	1	2.95	52.56	0.0002
C	0.8256	1	0.8256	14.7	0.0064
AB	0.3422	1	0.3422	6.09	0.0429
AC	0.09	1	0.09	1.6	0.2461
BC	0.9702	1	0.9702	17.27	0.0043
A2	17.61	1	17.61	313.46	<0.0001
B2	20.43	1	20.43	363.6	<0.0001
C2	33.25	1	33.25	591.84	<0.0001
Residual	0.3932	7	0.0562	0.5942	0.6512
Lack offit	0.1212	3	0.0404		
Pure error	0.272	4	0.068		
Cor Total	85.8	16			
R^2^ = 0.9954; Adjusted R^2^ = 0.9895; Predicted R^2^ = 0.9724					

**Table 7:** Variance analysis and significance tests of MS-7.

Source	Sum of Squares	Df	Mean Squares	F-Value	*p*-Value
Model	97.54	9	10.84	310.37	<0.0001
A	0.0561	1	0.0561	1.61	0.2455
B	7.68	1	7.68	220.04	<0.0001
C	0.3081	1	0.3081	8.82	0.0208
AB	0.0225	1	0.0225	0.6444	0.4485
AC	0.5112	1	0.5112	14.64	0.0065
BC	2.04	1	2.04	58.56	0.0001
A2	9.52	1	9.52	272.67	<0.0001
B2	33.28	1	33.28	952.99	<0.0001
C2	35.75	1	35.75	1023.75	<0.0001
Residual	0.2444	7	0.0349	2.3	0.2196
Lack offit	0.1546	3	0.0515		
Pure error	0.0898	4	0.0224		
Cor Total	97.78	16			
R^2^ = 0.9962; Adjusted R^2^ = 0.9914; Predicted R^2^ = 0.9739					

## Data Availability

The original contributions presented in the study are included in the article, further inquiries can be directed to the corresponding author.
